# A Case of Reynaud’s Disease

**Published:** 1887-01

**Authors:** T. B. Adam


THE CHICAGO

MEDICAL JOURNAL AND EXAMINER.

VOL. LIV. JANUARY, 1887. No. 1.

### ORIGINAL COMMUNICATIONS.

### A CASE OF REYNAUD'S DISEASE IN A CHINESE GIRL, TERMINATING FATALLY. *By* T. B. ADAM, M.D.

[Read before the United States Naval Medical Society, Washington, December 2d, 1886.]

The case recorded below interested and puzzled me much while under observation. It was not till opportunity was afforded, during a holiday trip home, of consulting my former teacher, Dr. Finlayson, that I learned the name and real nature of the disease from which my patient suffered.

An interesting account of Reynaud's disease, with illustrative cases, is published by Dr. Southey in the St. Bartholomew's Hospital Reports, vol. xvi, 1880. Drs. Finlayson, Barlow and many others have also reported cases.

My case is a well-marked example of the severest form of the disease. The association of fever, with the periodicity displayed in the attacks of "local asphyxia," and apparent improvement during the administration of quinine, would suggest malarial poison as the possible cause in this particular case, of the "exaggeration of the excito-motor power of the cord in presiding over the vaso-motor centers."

The following notes were taken at the bedside before I had read any account of the disease:

Tieng Tie—a female child, aged eight, residing in the city of Foochow, China—admitted to Foochow Native Hospital in the beginning of March, 1885, suffering from advanced gangrene of both feet. Patient's grandfather states that from birth the child was weakly. Suffered much in early childhood from scabies and boils. Never had small-pox.

When six years old the feet were bound according to Chinese custom. Cried much thereafter; but grandfather denies that any marked deterioration of health resulted.

Present ailment began three months before admission to hospital. While walking out one day the child complained bitterly of much pain in the calf of the left leg. On examination, the skin over calf was found bluish-black in color and cold to the touch. The child was allowed to go about at will, and in four days the discoloration entirely disappeared. A month later the child was vaccinated on the arm. The vesicles ran a normal course, leaving three good scars. One month before admission to the hospital, after partaking of some native wine, according to the grandfather's statement, the child became sick and feverish, and had to lie down in bed. On the following day exquisite pain was complained of in the left foot and lower part of the leg. The child screamed out in agony, and after a short time the left foot was observed to become swollen, black in color and perfectly cold. During the course of the following day the right foot became similarly affected, the child being again in agony with pain for an hour or two. The circulation in both feet was at once completely and finally arrested, and gangrene was rapidly established. A week later, in the forenoon, the child complained of a tingling in both ears. On examination, the ears were found to be blue and cold, while on either cheek bluish-black patches were present. After an hour the parts affected resumed their normal appearance. On the forenoon of the following day the ears and cheeks again were similarly affected, the seizure terminating in about an hour. The day after, likewise in the forenoon, the right hand became extremely painful, bluish-black discoloration appeared, and the hand felt quite cold to touch. Normal circulation was restored within an hour. A day or two later the left hand was similarly attacked.

From then till now the child's history has been that, on the forenoon of every day, either ears and cheeks, or one or both hands have been subjected to attacks of temporarily arrested circulation, with imminent gangrene, yielding, however, after an hour or more, and normal circulation being gradually restored. The ears or cheeks were never observed to be affected on the same days as the hands, but alternately.

The right hand has suffered most, having been attacked in all ten times before patient's admission to hospital.

Family history—Patient's father died at the age of twenty-eight. He suffered all his life from poor health, having cough and spit. Was an opium-smoker. The immediate cause of his death was chronic dysentery. Mother is alive, aged thirty. Suffers from chronic dyspepsia. Has no cough. Three children alive; one lost—a four months' miscarriage. Patient is the oldest child. The second, aged five, suffers from cough and attacks of diarrhœa. The youngest, a boy of two, apparently enjoys good health. No admission of syphilis is made of either parent.

Present condition—Patient is a poor, feeble looking child, with sallow complexion. Her breath has a distinctly gangrenous odor. The feet and lower third of both legs are perfectly black and gangrenous. Toes are shriveled up and dry.

An irregular line of demarcation exists about the junction of the middle and lower thirds of both legs, and separation has already considerably advanced. The left leg is more extensively affected than the right.

The child is very nervous and irritable, her face wearing an expression of having suffered much.

March 11th. Since admission the attacks of local congestion have recurred daily. The native assistant in hospital reports that the seizures occur daily with great regularity, between the hours of 11 A.M. and noon. Latterly the hands have been more frequently affected than ears or cheeks. This morning my visit was made a little before 11 A.M. The child was then lying quietly in bed, her hands looking normal. Taking a seat at bedside, after a short time of waiting, I observed both hands becoming congested. The nails first became blue and the fingers cold. Gradually the congestion deepened, fingers were swollen and almost perfectly black. The backs of both hands became bluish-black in color, and for an inch or two up the forearms mottling appeared. The radial pulse was just perceptible. The child screamed loudly with pain, evidently paroxysmal in character, as she would be quiet for a few minutes, answering questions readily, and then suddenly burst forth in shrieks. The right hand was much more deeply affected than the left. The pain seemed to follow upon, not precede, the arrest of the circulation. Both hands became quite cold to the touch, and sensibility was much diminished. Gentle rubbing upward toward trunk was tried to promote venous return, but without result. In about half an hour from the commencement of the seizure, both hands to all appearance seemed in a state of gangrene. I remained over an hour at the bedside, and just before leaving evidence was afforded of the circulation being restored in left hand. Right hand remained in state of gangrene. The hands were wrapped up in cotton wool. Heart sounds were perfectly normal. The child's restless condition prevented the use of the thermometer, but the skin of body felt normally cool to the touch.

March 14th. Since the 11th inst. the right hand has remained in the condition described in the last note. The fingers are quite black, the middle and little fingers at extremities shrunk in a state of complete dry gangrene. The whole hand is swollen and feels cold. The left hand recovered its circulation perfectly, and though it has been subjected to two fresh attacks since, it looks, at my visit this morning, quite normal. While I was in the ward, 11:30 A.M., the left hand was again attacked. During the seizure the right hand was again involved, the swelling in it becoming greater and pain being present. After cessation of the acute pain the temperature was taken in the mouth; it was 99.4°F. No rigors or sensations of cold have ever been experienced. The child's breath has a very marked gangrenous odor. The finger of the right hand was pricked with a needle. No pain felt; no flow of blood. Blood was easily obtained from the finger of the left hand. Examination under microscope showed no *filariæ sanguinis hominis* present. Child's general condition is fair, but a tendency to diarrhœa occurs, necessitating free administration of bismuth. Yesterday, quinine in one grain doses every three hours was prescribed.

March 15th. Since yesterday's seizure the circulation has not been restored to fingers of left hand, the tips of middle and ring fingers have shrunk. The left hand is swollen and bluish-red in color. Right hand and lower part of forearm still remain much swollen and discolored. The feet, which have been dressed with carbolic oil twice daily, are rapidly undergoing natural amputation. The urine examined this morning is found to contain albumen. Under microscope pus cells and broken down granular corpuscles are found; also a few granular tube casts; no filariæ.

March 16th. A marked improvement has to be noted to-day. No fresh seizure either in hands or ears has occurred since the 14th inst. To my great surprise, the right hand, which I had deemed hopelessly committed to gangrene, has all but completely recovered, the swelling gone, and normal circulation restored throughout the hand and thumb. The terminal portions of the forefingers are black and shrunken. The left hand also has recovered its circulation save in the extreme tips of the middle and ring fingers, which are black and gangrenous. The child's general condition is improved and she is fairly cheerful. Temperature normal.

March 17th. General state of patient not so good. On visit at noon temperature in mouth was 101°F., pulse 140. No fresh seizures in hands or ears.

March 18th. This morning the left ear was observed slightly congested. Quinine increased to three grains every four hours. The gangrenous odor of breath has all but disappeared.

March 20th. Since last note there has been no fresh seizure. Pain in belly is much complained of and diarrhœa persists. Santonine, three grains given.

March 23rd. No fresh seizures now since the 18th. Pain in belly still complained of and diarrhœa persists. Four or five lumbrici were passed this morning.

March 24th. Both feet dropped off this morning, separation having taken place through the ankle joints. Lower parts of tibiæ and fibulæ protrude through soft tissues of stumps. Child is much exhausted.

March 25th. No improvement in general state. Slight tonsilitis present.

April 2nd. Stumps look fairly well. No fresh seizures to report. General exhaustion is more marked and diarrhœa continues. Two drachms of port wine given every two hours

April 6th. Patient has gradually become weaker. Pains in belly of an intermittent character have been persistently complained of.

April 7th. Patient died last night, apparently from pure exhaustion. No post mortem examination permitted.

NOTES ON REYNAUD'S DISEASE.

Allied to the every day complaint of numb or dead fingers. A distinction made between the milder form of "local syncope" and the more serious form of "local asphyxia."

Local asphyxia—circulation comes to a stop with blood retained within the capillaries; blood does not pass, but veins remain full and part assumes a black appearance.

Probably no oxidation takes place, or only very imperfect nutritional chemical interchange, and therefore the blood will not pass through the capillaries. Part affected is numb, cold and has lost tactile sense and local sensibility—does not bleed when pricked. A sharp, burning pain is complained of. Degree of pain suffered bears relation to depth of color of affected skin area, being most severe when fullest of black blood.

No general symptoms of importance beyond the local pains.

Tendency of local asphyxia and local gangrene to be symmetrical on the two sides of the body.

Reynaud's definition of the disease: "A neurosis characterized by an exaggeration of the excito-motor power of the cord in presiding over the vaso-motor centers."

Of complaints resembling Reynaud's disease (but still very distinct) Englisch enumerates ergotism, frost-bite, diabetic gangrene, nephritis interstitialis, the cyanosis of heart disease, syphilis and lepra.

As hereditary causes may be placed all such as disturb the vaso-motor system, brain injuries, mental shocks, frights, concussions, etc.

Reynaud attributes the local mischief to cramp-like contractions of the arterio-capillaries. He thought quinine, strychnine and electricity appropriate remedies.

He regards the disease as an illustration of spinal vaso-motor reflex, of which he perceives three grades or degrees:

I.—Slight reflex spasm of arterioles—local syncope.

II.—Profound arrest of circulation by reflex spasm—local asphyxia.

III.—Mortification by prolonged arrest of circulation; by spinal excito-motor spasm.

The symmetrical nature of the lesion is due to the common central spinal vaso-motor regulator center.

Further, clinically we perceive three stages of symmetrical gangrene:

I.—Of invasion—local asphyxia with imminent gangrene (lasting about a month).

II.—Of gangrene—access and duration; whole mortification limited and complete within ten days, usually.

III.—Of healing by suppuration and granulation, in a period varying from twenty days to ten months.

### ADDENDUM.

Extract from a letter received from Surgeon G. W. Woods, U.S.N., transmitting the report of Dr. T. B. Adam:

"I herewith inclose a singularly interesting report by

Dr. T. B. Adam of a case of Reynaud's disease, in which I was much interested while at Foochow, China, in the winter and spring of 1885. I send it to you for the purpose of presentation to the Naval Medical Society when a favorable opportunity offers. You will find an exhaustive account of this disease in the fifth volume of the 'System of Practical Medicine,' published by Lea Brothers & Co., and edited by Wm. Pepper, M.D., LL.D.; the concluding volume just out. It is treated under the head of 'Symmetrical Gangrene,' or 'Local Asphyxia,' and Reynaud is given credit of first studying it in 1862, when he reported twenty-eight cases; and Billroth, Weir Mitchell, Mills, A. McLane Hamilton and J. C. Warren, as well as others, have since reported cases and discussed the subject.

"The pathology of the disease seems to indicate its origin to be at times centric, at others reflex, affecting the vaso-motor centers and producing symmetrical spasms of the arterial and venous systems at their terminal distribution, and, when reflex, may be caused by irritative conditions of the skin, brain, and viscera, especially the female genitals. It is most commonly a disease of adult life, more common in the male than female, and may be caused by cold, the various forms of exhaustion, anæmia, mal-nutrition, mental agitation and uterine disorders, though in some cases no cause has been determined.

"The most satisfactory treatment has been tonics, massage, elevation of the limbs, and wrapping them in cotton batting, with the application of antiseptic lotions, if necessary. Reynaud used a galvanic current, descending through spinal cord, with asserted success. Others have failed to notice any benefit from galvanism."

